# Efficient Amplification of Chimeric Adenovirus 5/40S Vectors Carrying the Short Fiber Protein of Ad40 in Suspension Cell Cultures

**DOI:** 10.1371/journal.pone.0042073

**Published:** 2012-07-31

**Authors:** Marta Miralles, María Mercedes Segura, Meritxell Puig, Assumpció Bosch, Miguel Chillon

**Affiliations:** 1 Center of Animal Biotechnology and Gene Therapy (CBATEG), and Department of Biochemistry and Molecular Biology, Universitat Autònoma de Barcelona, Barcelona, Spain; 2 Institut Català de Recerca i Estudis Avançats (ICREA), Barcelona, Spain; St. Louis University, United States of America

## Abstract

The human adenovirus 40 (Ad40) is a promising tool for gene therapy of intestinal diseases. Since the production of Ad40 *in vitro* is extremely inefficient, chimeric Adenovirus 5/40S vectors carrying the Ad40 short fiber on the Ad5 capsid have been developed. However, Ad5/40S productivity is low. We hypothesized that low productivity was a result of inefficient viral entry into producer cells during amplification. To this end, we have developed a production strategy based on using 211B cells (expressing Ad5 fiber) during amplification steps, while Ad5/40S infectivity is further improved by adding polybrene during infections. In addition, the optimal harvesting time was determined by evaluating the Ad5/40S viral cycle. The developed production strategy significantly reduces the number of amplification cycles and duration of the process. Finally, to further facilitate Ad5/40S production, 211B cells were adapted to suspension thus allowing to easily upscale the production process in bioreactors.

## Introduction

Human Adenovirus 40 serotype (Ad40) is an enteric adenovirus of the subgroup F. This adenoviral serotype is an important etiologic agent of gastroenteritis in children [1]–[3]. Due to its enteric tropism, vectors derived from Ad40 constitute interesting candidates for gene therapy of intestinal diseases such as Crohn’s Disease. Initial attempts to grow Ad40 in HeLa or other cell lines commonly used to isolate and propagate intestinal viruses from patients stool samples were unsuccessful. This led to the idea that Ad40 viruses were unable to grow *in vitro*. However, subsequent experiments revealed that Ad40 could grow in some cells, for instance HEK293 cells, enabling its propagation in the laboratory [4]–[6]. Although the Ad40 is able to infect HEK293 cells, its production *in vitro* is very inefficient, resulting in low titers in comparison to other adenoviral serotypes [7], [8].

On the other hand, vectors derived from adenovirus type 5 (Ad5) are widely used in human clinical trials (Journal of Genetic Medicine Website, www.wiley.co.uk/genmed/clinical). Ad5 first interaction with the host cell is through the binding of the fiber protein and the coxsackievirus and adenovirus receptor (CAR). Since CAR is widely distributed on the cell surface of many cell types, Ad5 vectors display a broad tropism [9]–[12]. In order to restrict vector tropism, chimeric Ad5 vectors containing the fiber protein of other adenoviral serotypes have been developed [13], [14]. In fact, the possibility of infecting host cells by CAR-independent entry pathways is an interesting tool to limit the characteristic broad tropism of Ad5 viruses [15]. For instance, it has been described that Ad40 contains two different fibers: a long one (F40L) and a short one (F40S) [10], [16], [17]. Only the long fiber binds CAR while the short fiber is believed to be responsible for the enteric tropism [18]. In this regard, the generation of chimeric Ad5/40S mutants (Ad5 capsid with the F40S protein) has shown to ablate CAR binding while conferring a novel tropism to Ad5 viral vectors, and thus, intravenous administration of Ad5/40S vectors resulted mainly in liver and spleen transduction, as shown by the presence of viral DNA and transgene expression in these organs, while the virus was hardly detected in the intestine [15]. However, and contrary to the reduced affinity of Ad5/41S vectors for human intestinal epithelium [19], when given directly into the gastrointestinal tract by rectal administration in vivo, chimeric Ad5/40S vectors mantain the enteric tropism [20].

Interestingly, Lu and collaborators [21] have recently reported efficient amplification of Ad41 vectors (another enteric adenovirus of the subgroup F) by using a new producer cell line expressing E1B55K from Ad41. However, although genomes from both, Ad40 and Ad41 viruses have been sequenced, the oncogenic potential of their proteins is unknown. Since there is leaky expression from viral genes from recombinant adenovirus vectors [22], the use of Ad40 and Ad41 as gene therapy vectors in humans should be restricted for biosafety reasons.

To address both, the difficulty of Ad40 vector production and the biosafety concerns, the use of chimeric Ad5/40S vectors, combining the capsid structure of fully characterized Ad5 vectors and enteric tropism mediated by the F40S fiber proteins, is attractive. Various production protocols to amplify chimeric Ad5 vectors including the short fiber proteins F40s and F41S have been reported [7], [8] [23]–[25] Unfortunately, although the production of the chimeric adenoviral vector Ad5/40S *in vitro* is more efficient than that of wild type Ad40, the purity and the productivity per cell is still not sufficient to achieve the desirable viral titres. In this work, we describe the development of a new production protocol that allows fast and scalable production of Ad5/40. The production strategy has been optimized by i) studying the Ad5/40S viral cycle to determine the optimal harvesting time, ii) using the 211B cell line for vector amplification, iii) adapting the producer cell line to grow in suspension culture and low serum media, iv) improving infection conditions using polybrene, v) reducing the production time.

## Results

### Adaptation and Characterization of 211B Cells to Suspension Culture

In order to address the inefficient infection of Ad5/40S vectors in HEK-293 cells, we selected 211B as producer cells. This cell line derives from HEK-293 cells and constitutively expresses the fiber protein from Ad5 (F5) [26], [27]. Ad5/40S production in 211B cells will generate mosaic virions containing both F5 and F40S fiber proteins. Fiber mosaicism should improve the infectivity of the Ad5/40S virions during the amplification cycles by allowing a more efficient entry mediated by CAR-F5 interaction on 211B producer cells.

Adherent 211B cells were routinely cultured in DMEM (Dulbecco’s Modified Eagle’s Medium) media supplemented with 10% fetal bovine serum (FBS). To allow scalability of the process [28], we adapted the 211B cell line to grow in suspension and low protein medium. To this end, culture medium DMEM containing 10% FBS was sequentially substituted by a serum-free growth medium (SFMII) in the presence of a low dose of FBS (1% FBS or 0.5% FBS) or no FBS at all. Addition of 0.5% FBS allowed better cell growth and viability in comparison with cultures grown in the absence of FBS, while addition of 1% of FBS led to the formation of large cell aggregates (data not shown). The cells newly adapted to low-protein suspension conditions were named 211BS. 211BS cells were transferred into shake flasks at a density of 7×10^5^ cells/ml and kept in suspension at 110 rpm. After 18 serial passages, adapted cells grew individually or in small aggregates of 4–5 cells, and up to a density of 2.5×10^6^ cells/ml.

**Figure 1:**
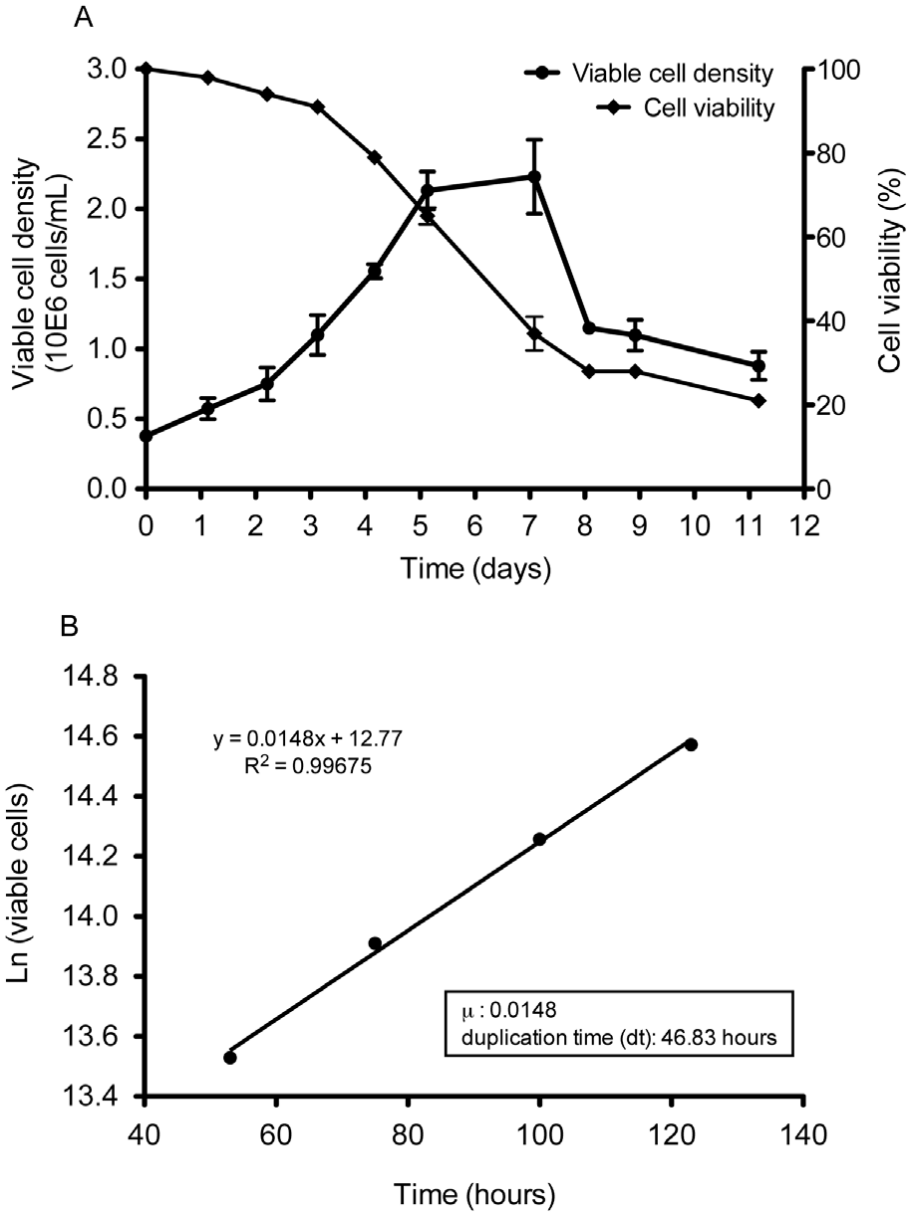
Characterization of 211BS cells. (A) Cell growth curve and viability of suspension-adapted 211BS cells. (B) Duplication time of the suspension-adapted cell line.

To determine the kinetics of growth of 211BS cells, cells were seeded in triplicate at 3.5×10^5^ cells/ml. Viability and cell density were evaluated daily. As observed in the growth curve (Figure 1A), the viability of 211BS cells was over 80% during exponential cell growth. Cells grow to high densities (∼2.5×10^6^ cells/ml) in batch mode. The µ (growth constant) was 0.0148 (Figure 1B) implying a duplication time of 46.83 hours.

**Figure 2:**
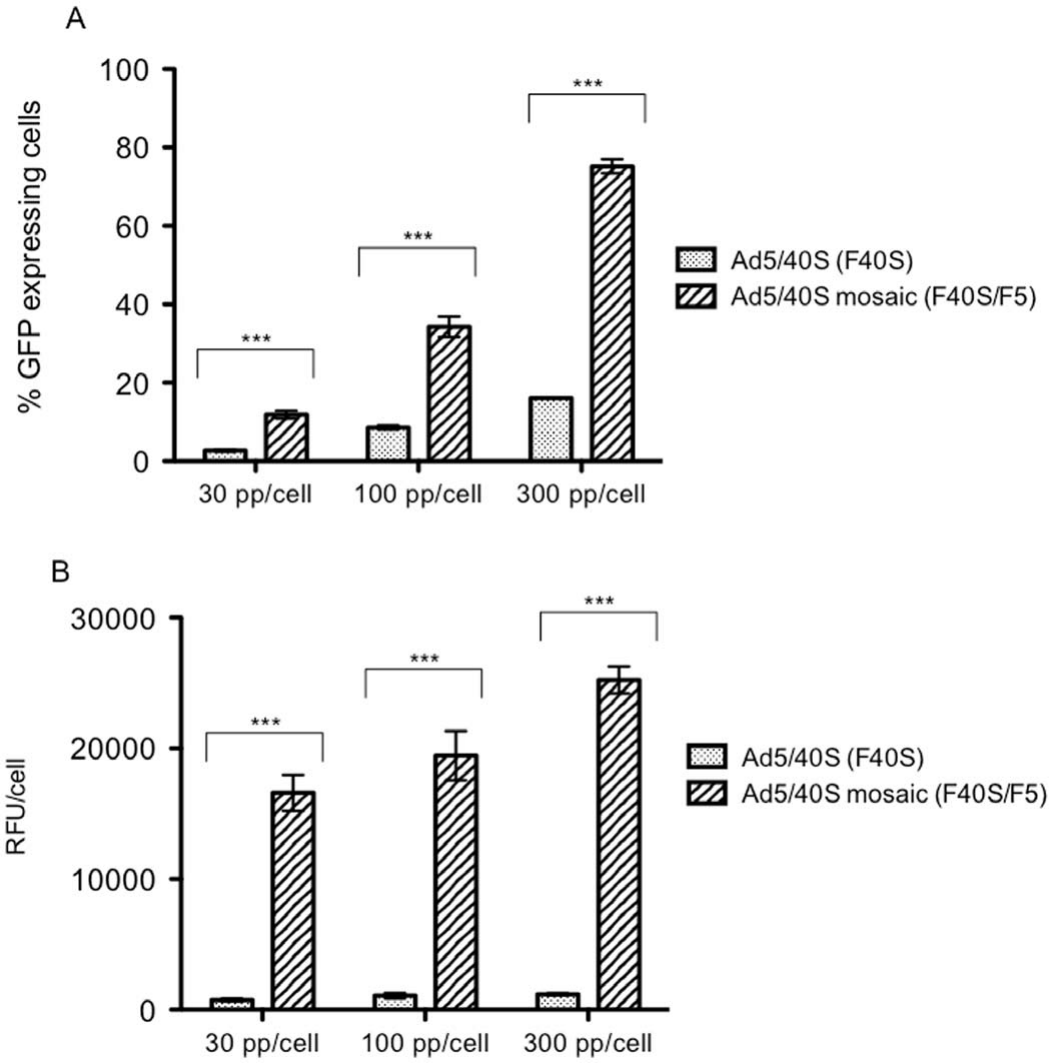
Analysis of the infection efficiency of mosaic-chimeric Ad5/40S-CMV-GFP vector. Percentage of GFP expressing cells (A); and quantification of relative GFP expression per cell (B). The infection was performed in cells 293F using 3 different concentrations of the Ad5/40S vectors (30, 100, 300pp/cell). Values presented are shown as mean ± Standard Deviation using n = 8 for each condition. Statistical comparisons between groups were made by two-ways ANOVA with a post hoc Bonferroni test for multiple comparisons (*** p-value <0.001).

Once 211BS cells were adapted to grow in suspension and low serum concentrations (0.5%), we proceeded to demonstrate that the mosaic-chimericAd5/40S vectors produced with these cells (displaying both F40S and F5 fibers on the capsid) could infect cell lines expressing the CAR receptor better, and thus produce better yield when propagated on such cells. For this purpose, 293F cells (CAR-expressing cells) were infected with different concentrations (30, 100 and 300pp/cell) of Ad5/40S vectors (Figure 2). Results have shown that mosaic/chimeric Ad5/40S generated in 211BS cells infected 4 to 5 times more the 293F cells (p-value <0.001) than chimeric Ad5/40S, regardless the condition used, indicating that the presence of Ad5 fibers helps mosaic vectors to infect 293F cells.

### Polybrene Improves Chimeric Adenovirus 5/40S Infection in 211BS Cells

Cationic polymers such as polybrene are well known enhancers of retrovirus and lentivirus vectors gene transfer efficiency [29]–[31]. Polybrene increases retrovirus transduction by enhancing receptor-independent virus adsorption on target cell membranes. We hypothesized that addition of polybrene during amplification cycles would also facilitate the entry of Ad5/40S particles into producer cells. In fact, cationic polymers like polybrene were also reported to interact with negatively charged Ad5 capsids facilitating their interaction with the cell membrane [32]; [33]. However, when adenovirus particles have a more neutral charge, as it happens to CAV-2, cationic molecules (including polybrene) do not interact well to virions [34]. Of note, the tail and shaft domains of the F40S protein have a high content of basic amino acids, which results in a pI of 9.1 compared to a pI of 6.1 for Ad5 fiber protein (pI values were calculated by the EMBL WWW Gateway to Isoelectric Point Service). Based on the above, the overall negative charge of Ad5/40S particles should be lower than that of Ad5 particles. Therefore, it was unclear whether polybrene would have an effect on the chimeric adenovirus entry to target cells.

**Figure 3:**
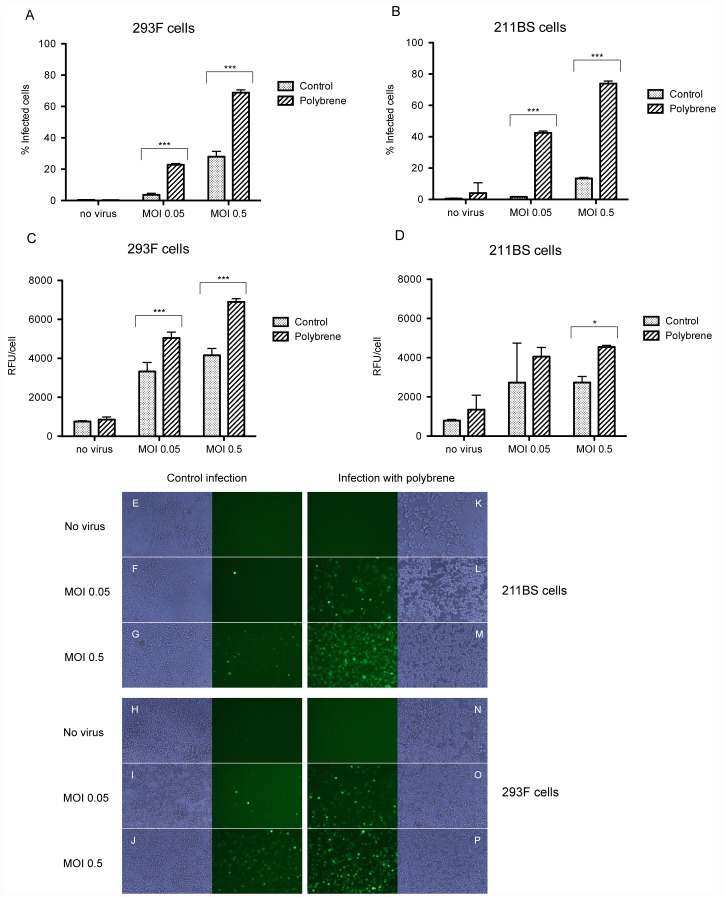
Analysis of the infection efficiency of Ad5/40S with polybrene. Percentage of Ad5/40S-CMV-GFP infection in 293F (A) and 211BS cells (B) using two different doses (MOI 0.05 and 0.5. Quantification of the GFP expression (RFU/cell) in 293F (C) and 211BS cells (D). E-P: GFP expression of Ad5/40S in 211BS and 293F infected cells in presence (E–J) or absence (K–P) of polybrene. Results are the average two independent experiments performed in triplicate. Statistical comparisons between groups were made by two-ways ANOVA with a post hoc Bonferroni test for multiple comparisons (*** p-value <0.0001; * p-value <0.05).

To test the effect of polybrene on chimeric adenovirus infection, 293F and 211BS cells were infected with increasing MOI’s of Ad5/40S-CMV-GFP, in presence or absence of polybrene (9 µg/ml or 0 µg/ml, respectively). The percentage of infected cells and the level of the GFP expression per cell were determined by FACS analyses. As observed in Figure 3, polybrene significantly enhances both, the percentage of infected 293 and 211BS cells (A-D, E-P) and the gene expression per cell (C, D), while cell viability seems to be not affected.

### Study of Ad5/40S Viral Cycle in 211BS

**Figure 4:**
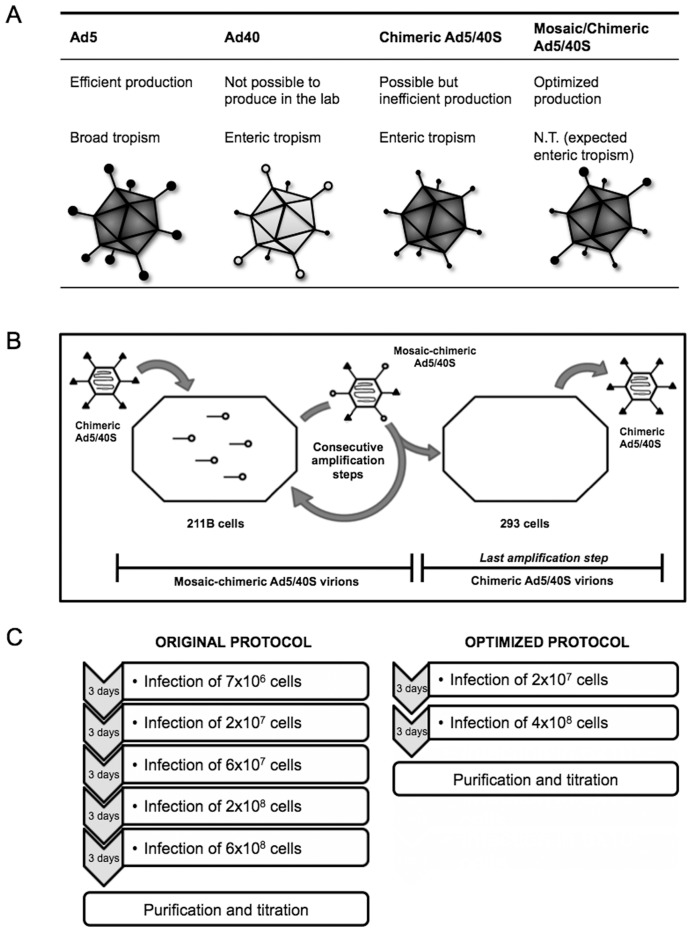
Diagrams of the chimeric amplification process. (A) Comparative diagram of Ad4, Ad40, chimeric Ad5/40S and mosaic-chimeric Ad5/40 vectors. N.T: Not Tested. (B) Amplification strategy of Ad5/40S vectors. The first amplification step is performed by infecting 211BS cells with the chimeric Ad5/40S and polybrene. Intermediate steps are performed also in 211BS cells by using mosaic-chimeric Ad5/40S and polybrene. Last step is performed in 293F cells to obtain chimeric Ad5/40S vectors. (C) Comparative diagram of duration and cell-scale.

Ad5/40S produced by 211BS cells are expected to have both, F5 and F40S proteins (mosaic-chimeric Ad5/40S) whereas Ad5/40 produced by 293F cells should only display F40S on their surface (chimeric Ad5/40S) (Figure 4A). In order to maximize productivity of Ad5/40S, we envisioned a production scheme in which amplification of the chimeric vector is performed in 211BS (Figure 4B). However, in order to obtain pure chimeric (not mosaic) Ad5/40S particles, the last step of amplification needs to be performed in 293F cells.

**Figure 5:**
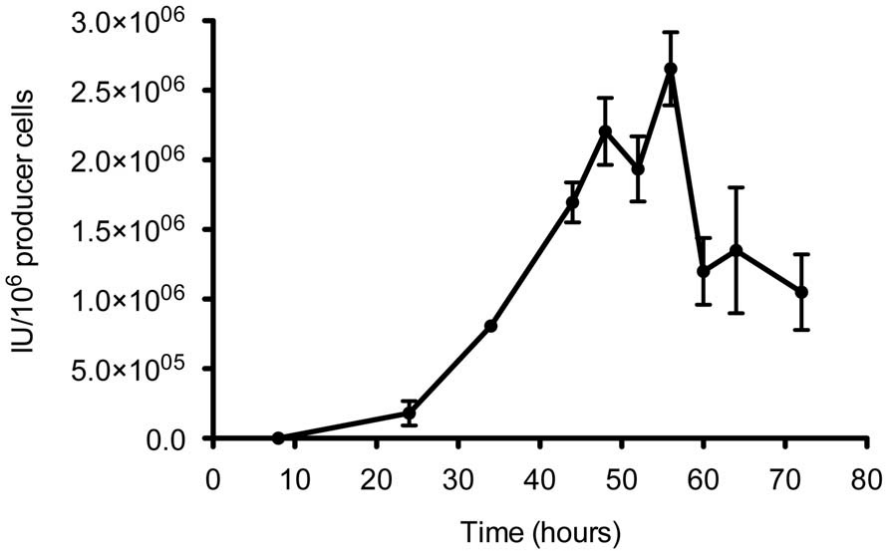
Virus cell cycle of mosaic-chimeric Ad5/40S vectors. 211BS cells were infected with Ad5/40S-GFP at MOI = 0.5. Samples were collected every 4 hours between 44 h and 64 h post-infection. Productivity is measured in total infection units produced per 10^6^ cells.

We had previously reported that chimeric adenovirus Ad5/40S had a viral cycle between 48 and 60 hours [20]. To more accurately determine the optimal harvesting time, a more precise analysis of Ad5/40S viral cycle was performed. In this study, viral titers were measured every 4 hours between 44 and 64 hours post-infection. In addition, we worked at a low MOI (0.5) to avoid saturation and displacement of the curve associated to the entry of several particles per cell, which may accelerate the virus cycle. As it can be observed in Figure 5, at a MOI of 0.5, the main production peak is at 56 hours post-infection.

### Vector Ad5/40S Production with the Optimized Protocol

To confirm the strength of the developed production strategy, suspension growing 211BS cells were infected with Ad5/40S-CMV-GFP at a MOI of 1 in the presence of polybrene. At 56 hours post-infection cells were harvested, lysed by freeze-thaw and cell debris was removed by centrifugation. The resulting lysate was used to infect 4×10^8^ 293F cells. Fifty six hours post-infection, cells were harvested and centrifuged to separate cell pellet and supernatant. Since the Ad5/40S-CMV-GFP virus contains the Death Protein (ADP) gene, which may facilitate virus release into the supernatant by cellular lysis [35], supernatants were concentrated by ultrafiltration using a hollow fiber utrafiltration system. Subsequently, adenovirus particles from both, the cell pellet and the concentrated supernatant were purified by CsCl gradients followed by gel filtration. Viral titers in physical particles and infectious particles have an average yield of 0.85×10^12^ pp and 3.8×10^9^ IU, for the original protocol, and 1.75×10^12^ pp and 6.9×10^9^ IU, for the optimized protocol (Table 1), with an average productivity around 250pp/cell. As observed in Figure 4C, the number of viral amplification steps required is considerably lower in the optimized protocol vs. the original protocol; which translates into the reduction of consumables and time needed for vector production.

**Table 1:** Comparative table between the original and newly optimized protocols.

	Originalprotocol(n = 5)	Optimizedprotocol(n = 8)
Time in obtaining harvest	15 days	6 days
Number of amplification steps	5	2
Number of cells	2×10^8^	2×10^8^
Necessary growth medium	180 mL	200 mL
Titration (Infection Units in 293 cells)	3,8×10^9^ IU	6,9×10^9^ IU
Titration (Physical Particles)	0,85×10^12^ pp	1,75×10^12^ pp

## Discussion

One of the key factors for successful gene therapy is to have a vector that efficiently and selectively infects target cells, thereby minimizing the side effects associated with transgene expression in unwanted cells. Previous results reported by our group showed that chimeric Ad5/40S vectors display a marked intestinal tropism likely attributable to the Ad40 short fiber protein [20]. These chimeric vectors could be used for gene therapy of diseases affecting the gastrointestinal tract. However, Ad5/40S vectors cannot be efficiently amplified, probably because of the absence or low abundance of the primary receptors for F40S in the permissive producing HEK-293 cells, resulting in poor virus production.

In order to improve Ad5/40S viral titers, we have designed an optimized production strategy using 211B producer cells. These cells constitutively express F5, and therefore upon infection with Ad5/40S, fiber mosaic virus particles (displaying both F5 and F40S on their capsids) are formed. The 211B cell line was originally used to retarget non-Ad5 or fiberless-Ad5 particles in order to easily and rapidly change their tropism in vivo [26]. In this work, we have used this retargeting strategy to facilitate re-entry of the mosaic-chimeric Ad5/40S virions during amplification steps, through interaction between the F5 fiber protein of the virus capsid and the CAR receptors on 211B producer cells. An attractive aspect of this dual strategy is its flexibility, since it can be used to amplify any chimeric Ad5 vector by substituting the F5 protein with the fiber protein from another serotype, especially when the chimeric vector does not efficiently infect permissive HEK-293 cells.

To further facilitate the entry of Ad5/40S particles into producing cells we have also added polybrene during amplification cycles. One of the major advantages of polybrene is that it can be added directly to the media, as it does not require previous formation of complexes with pure adenovirus particles, thus avoiding the need for purification of the vector during the amplification process. Of note, polybrene-mediated enhancing effects on adenovirus infection are only observed when using Freestyle serum-free medium, whereas SFMII medium completely blocks the effect of polybrene (data not shown). Interestingly, the same trend is observed with other cationic molecules such as polyethilenimine (PEI) used for transient transfection [36], suggesting the presence of compounds in SFMII serum-free medium that may counteract with cationic polymers (i.e. negatively charged polymers such as heparin or dextran sulphate typically added to commercial media to keep cells in suspension). Last we have also analyzed the viral cell cycle of chimeric Ad5/40S vectors and determined 56 hours after infection as the most appropriate time to stop the production process and harvest the virus.

In summary, a new chimeric Ad5/40S production procedure has been developed in this work. The strategy is based on the use of cultures in suspension to allow the scalability of the production process, as well as, on increasing chimeric Ad5/40S infectivity towards producer cells and consequently, allowing a higher productivity per cell, from an initial amplification factor per step of ×3–4 up to ×20–25 times as observed now. This allows reducing the number of amplification steps, which carries several advantages such as minimization of total processing time of virus production, reduction of consumables, and most importantly, it decreases the risk of generating replication competent virions by recombination of vector sequences with E1 sequences present in the cells used for production.

## Materials and Methods

### Virus Stock Generation

Ad5/40S-GFP-CMV was obtained from Dr. Hirofumi Hamada (Sapporo Medical University). Adherent HEK-293 cells (Q-BIOgene, Montreal, Canada) were grown in DMEM medium (E15-810, PAA laboratories, Linz, Austria) supplemented with 10% fetal bovine serum (FBS) (PAA laboratories, Linz, Austria) and Penicillin (100 U/ml)/Streptomycin (0,1 mg/ml) (PAA laboratories, Linz, Austria). Viral stocks were generated by infection and sequential amplification in HEK 293 cell cultures grown in 15-cm plates until 30 plates were reached. Virus was purified by two consecutive rounds of CsCl isopycnic density ultracentrifugation and desalted using a Sephadex PD-10 column (Amersham Biosciences, Uppsala, Sweden) as previously reported [37]. In order to facilitate cloning of therapeutic genes into the Ad5/40S genome, we have adapted the procedure in bacteria and constructed a new plasmid (pER F40S) carrying the Ad5 genome but with the F40S gene instead of the F5 gene.

### 211B Adaptation to Suspension Culture

Adherent 211B cells [27] were cultured in DMEM supplemented with 10% FBS and Penicillin (100 U/ml)/Streptomycin (0,1 mg/ml). For adaptation to suspension culture and low protein conditions, this culture medium was gradually substituted throughout 8 culture passages with SFMII (11686-029, GIBCO), supplemented with 4 mM Glutamine (PAA laboratories, Linz, Austria), Penicillin (100 U/ml)/Streptomycin (0,1 mg/ml) and 1% Pluronics (24040-032, GIBCO). 211B cells in suspension (termed 211BS) were then transferred to 125 mL polycarbonate shake flasks at a density of 8×10^5^ cells/ml and kept in suspension by agitation in an orbital shaker at a speed of 110 rpm, 37°C and 5% CO_2_.

Growth kinetics of 211BS cells was evaluated in two independent experiments from passages 20 and 30. Cells were seeded at a density of 3.5×10^5^ cells/ml in shake flasks containing 20 ml of cell suspension. The experiment was run in triplicate. Viable and dead cells were counted daily for a period of 12 days. The specific growth constant (µ) corresponds to the slope of the fitted line during the exponential growth phase. The cell culture doubling time (td) was calculated as td  =  ln 2/µ.

### Effect of Polybrene on Ad5/40S Infectivity

211BS cells were seeded at a density of 1×10^6^ cells/ml in a final volume of 1 ml per well in 24-well plates and infected with Ad5/40S-CMV-GFP at two different MOI’s (0.5 or 0.05). Control cells were not infected. Infections were performed in the presence or absence of polybrene (9 µg/ml, as previously described [32], [38] (n = 4) in Freestyle serum-free medium (12338-018, Invitrogen) supplemented with Penicillin (100 U/ml)/Streptomycin (0,1 mg/ml) and 1% Pluronics (24040-032, Invitrogen). Cell cultures were supplemented with 0.5% FBS 4 hpi and harvested 30 hpi. After fixation with 2% paraformaldehyde, the percentage of GFP expressing cells was determined by FACS analysis (FACSCanto, Cytometry Service of Institute of Biochemistry and Biotechnology of UAB).

### Analysis of Ad5/40S cell cycle in 293 F cells

293F cells (11625-019, Invitrogen, Paisley, UK) were grown to a density of 1×10^6^ cells/ml in 125 mL shake flasks and infected with mosaic-chimeric Ad5/40S at a MOI of 0.5. The culture medium was replaced with fresh medium 8 hpi. Cells were transferred to 6-well plates (2 ml per well) and maintained at a speed of 110 rpm at 37°C and 5% CO_2_. At various points post-infection, cells were harvested (n = 5) and frozen at -80°C. Cell pellets were lysed by 3 freeze-thaw cycles and lysates titered. At 48 hours cells were fixed with 2% paraformaldehyde and the percentage of GFP expressing cells was assessed by FACS analyses.

### Vector Production Using the Optimized Protocol

211BS cells were grown to a density of 1×10^6^ cells/ml in 125 mL shake flasks (25 ml working volume) in Freestyle serum-free media supplemented with Penicillin (100 U/ml)/Streptomycin (0,1 mg/ml) and 1% Pluronics. Cells were infected with Ad5/40S-CMV-GFP at a MOI of 1. The infection was performed in presence of polybrene (9 µg/ml). Cell cultures were supplemented with 0.5% FBS 4 hpi and the percentage of GFP positive cells was estimated by fluorescence microscopy 30 hpi. Cell cultures were harvested 56 hpi and frozen at –80°C. Cells were lysed by 3 freeze-thaw cycles, centrifuged at 1620×g for 5 min to remove cell debris.

The last amplification cycle was performed in 293F cells grown to a density of 1×10^6^ cells/ml in 1L-shake flasks (200 ml working volume) in Freestyle serum-free media supplemented with Penicillin (100 U/ml)/Streptomycin (0,1 mg/ml) and 1% Pluronics. Cell cultures were infected by adding the cell lysate from the previous amplification step. The infection was performed in presence of polybrene (9 µg/ml). Cell cultures were supplemented with 0.5% FBS 4 hpi and harvested 56 hpi by centrifugation at 180×g during 5 min. The cell pellet was resuspended in 20 mL of supernatant and the remaining supernatant (∼190 ml) was stored separately at –80°C. The latter was concentrated down to 20 ml using a Midjet system (56-4110-25, Amersham Biosciences Corp., Westborough, MA, USA). The cell pellet was lysed by 3 freeze-thaws cycles, cell debris was removed by centrifugation at 1150 xg during 5 min and mixed with the previously concentrated supernatant. The crude viral stock was purified by double CsCl gradient, and chromatography using a molecular exclusion column as described above.

### Titration of Viral Physical and Infectious Viral Particles

Final purified viral stocks titers (physical particles/ml) were determined by optical density at 260 nm (1 OD260 unit = 1×10^12^ particles/ml), and infectivity (infectious units/ml) was measured by end-point dilution assay [39], [40]. Briefly, end-point dilution assay was performed by infecting HEK-293 cells with serially diluted virus samples in triplicate in the presence of polybrene (9 µg/ml). The number of transgene (GFP) expressing cells was determined 48–72 hpi by fluorescence microscopy.

## References

[1] Cunliffe NA, Booth JA, Elliot C, Lowe SJ, Sopwith W, et al (2010) Healthcare-associated viral gastroenteritis among children in a large pediatric hospital, United Kingdom. Emerg Infect Dis 16: 55–62. 20031043 10.3201/eid1601.090401 PMC2874353

[2] Uhnoo I, Wadell G, Svensson L, Johansson ME (1984) Importance of enteric adenoviruses 40 and 41 in acute gastroenteritis in infants and young children. J Clin Microbiol 20: 365–372. 6092424 10.1128/jcm.20.3.365-372.1984 PMC271331

[3] Wilhelmi I, Roman E, Sanchez-Fauquier A (2003) Viruses causing gastroenteritis. Clin Microbiol Infect 9: 247–262. 12667234 10.1046/j.1469-0691.2003.00560.x PMC7129320

[4] Brown M, Petric M, Middleton PJ (1984) Diagnosis of fastidious enteric adenoviruses 40 and 41 in stool specimens. J Clin Microbiol 20: 334–338. 6490823 10.1128/jcm.20.3.334-338.1984 PMC271324

[5] Brown M, Wilson-Friesen HL, Doane F (1992) A block in release of progeny virus and a high particle-to-infectious unit ratio contribute to poor growth of enteric adenovirus types 40 and 41 in cell culture. J Virol 66: 3198–3205. 1373207 10.1128/jvi.66.5.3198-3205.1992 PMC241087

[6] Hashimoto S, Sakakibara N, Kumai H, Nakai M, Sakuma S, et al (1991) Fastidious human adenovirus type 40 can propagate efficiently and produce plaques on a human cell line, A549, derived from lung carcinoma. J Virol 65: 2429–2435. 1826748 10.1128/jvi.65.5.2429-2435.1991 PMC240596

[7] Sherwood V, Burgert HG, Chen YH, Sanghera S, Katafigiotis S, et al (2007) Improved growth of enteric adenovirus type 40 in a modified cell line that can no longer respond to interferon stimulation. J Gen Virol 88: 71–76. 17170438 10.1099/vir.0.82445-0

[8] Tiemessen CT, Kidd AH (1994) Adenovirus type 40 and 41 growth in vitro: host range diversity reflected by differences in patterns of DNA replication. J Virol 68: 1239–1244. 8289359 10.1128/jvi.68.2.1239-1244.1994 PMC236569

[9] Bergelson JM, Cunningham JA, Droguett G, Kurt-Jones EA, Krithivas A, et al (1997) Isolation of a common receptor for Coxsackie B viruses and adenoviruses 2 and 5. Science 275: 1320–1323. 9036860 10.1126/science.275.5304.1320

[10] Roelvink PW, Lizonova A, Lee JG, Li Y, Bergelson JM, et al (1998) The coxsackievirus-adenovirus receptor protein can function as a cellular attachment protein for adenovirus serotypes from subgroups A, C, D, E, and F. J Virol. 72: 7909–7915. 10.1128/jvi.72.10.7909-7915.1998 PMC110119 9733828

[11] Tatsis N, Ertl HC (2004) Adenoviruses as vaccine vectors. Mol Ther 10: 616–629. 15451446 10.1016/j.ymthe.2004.07.013 PMC7106330

[12] Tomko RP, Xu R, Philipson L (1997) HCAR and MCAR: the human and mouse cellular receptors for subgroup C adenoviruses and group B coxsackieviruses. Proc Natl Acad Sci U S A 94: 3352–3356. 9096397 10.1073/pnas.94.7.3352 PMC20373

[13] Gall J, Kass-Eisler A, Leinwand L, Falck-Pedersen E (1996) Adenovirus type 5 and 7 capsid chimera: fiber replacement alters receptor tropism without affecting primary immune neutralization epitopes. J Virol 70: 2116–2123. 8642632 10.1128/jvi.70.4.2116-2123.1996 PMC190048

[14] Krasnykh VN, Mikheeva GV, Douglas JT, Curiel DT (1996) Generation of recombinant adenovirus vectors with modified fibers for altering viral tropism. J Virol 70: 6839–6846. 8794325 10.1128/jvi.70.10.6839-6846.1996 PMC190731

[15] Nakamura T, Sato K, Hamada H (2003) Reduction of natural adenovirus tropism to the liver by both ablation of fiber-coxsackievirus and adenovirus receptor interaction and use of replaceable short fiber. J Virol 77: 2512–2521. 12551989 10.1128/JVI.77.4.2512-2521.2003 PMC141073

[16] Kidd AH, Chroboczek J, Cusack S, Ruigrok RW (1993) Adenovirus type 40 virions contain two distinct fibers. Virology 192: 73–84. 8517033 10.1006/viro.1993.1009

[17] Tiemessen CT, Kidd AH (1995) The subgroup F adenoviruses. J Gen Virol 76 (Pt 3): 481–497. 10.1099/0022-1317-76-3-481 7897343

[18] Seiradake E, Cusack S (2005) Crystal structure of enteric adenovirus serotype 41 short fiber head. J Virol 79: 14088–14094. 16254343 10.1128/JVI.79.22.14088-14094.2005 PMC1280240

[19] Kesisoglou F, Chamberlain JR, Schmiedlin-Ren P, Kaz A, Fleisher D, et al (2005) Chimeric Ad5 vectors expressing the short fiber of Ad41 show reduced affinity for human intestinal epithelium. Mol Pharm 2: 500–508. 16323957 10.1021/mp0498897

[20] Rodriguez R, Romero C, Ferrer M, Burgueño JF, Gil G, et al (2006) Therapeutic Potential of the Chimeric Adenovirus 5/40 as Vector for Intestine-Directed Gene Therapy. Molecular Therapy 13: S5.

[21] Lu ZZ, Zou XH, Dong LX, Qu JG, Song JD, et al (2009) Novel recombinant adenovirus type 41 vector and its biological properties. J Gene Med 11: 128–138. 19097028 10.1002/jgm.1284

[22] Yang Y, Nunes FA, Berencsi K, Furth EE, Gonczol E, et al (1994) Cellular immunity to viral antigens limits E1-deleted adenoviruses for gene therapy. Proc Natl Acad Sci U S A 91: 4407–4411. 8183921 10.1073/pnas.91.10.4407 PMC43794

[23] Nicol CG, Graham D, Miller WH, White SJ, Smith TA, et al (2004) Effect of adenovirus serotype 5 fiber and penton modifications on in vivo tropism in rats. Mol Ther 10: 344–354. 15294181 10.1016/j.ymthe.2004.05.020

[24] Schoggins JW, Gall JG, Falck-Pedersen E (2003) Subgroup B and F fiber chimeras eliminate normal adenovirus type 5 vector transduction in vitro and in vivo. J Virol 77: 1039–1048. 12502819 10.1128/JVI.77.2.1039-1048.2003 PMC140814

[25] Schoggins JW, Nociari M, Philpott N, Falck-Pedersen E (2005) Influence of fiber detargeting on adenovirus-mediated innate and adaptive immune activation. J Virol 79: 11627–11637. 16140740 10.1128/JVI.79.18.11627-11637.2005 PMC1212603

[26] Von Seggern DJ, Huang S, Fleck SK, Stevenson SC, Nemerow GR (2000) Adenovirus vector pseudotyping in fiber-expressing cell lines: improved transduction of Epstein-Barr virus-transformed B cells. J Virol 74: 354–362. 10590124 10.1128/jvi.74.1.354-362.2000 PMC111546

[27] Von Seggern DJ, Kehler J, Endo RI, Nemerow GR (1998) Complementation of a fibre mutant adenovirus by packaging cell lines stably expressing the adenovirus type 5 fibre protein. J Gen Virol 79 (Pt 6): 1461–1468. 10.1099/0022-1317-79-6-1461 9634089

[28] Kamen A, Henry O (2004) Development and optimization of an adenovirus production process. J Gene Med 6 Suppl 1S184–192. 14978761 10.1002/jgm.503

[29] Davis HE, Rosinski M, Morgan JR, Yarmush ML (2004) Charged polymers modulate retrovirus transduction via membrane charge neutralization and virus aggregation. Biophys J 86: 1234–1242. 14747357 10.1016/S0006-3495(04)74197-1 PMC1303915

[30] Kaplan JM, Pennington SE, St George JA, Woodworth LA, Fasbender A, et al (1998) Potentiation of gene transfer to the mouse lung by complexes of adenovirus vector and polycations improves therapeutic potential. Hum Gene Ther 9: 1469–1479. 9681418 10.1089/hum.1998.9.10-1469

[31] Wang C, Pham PT (2008) Polymers for viral gene delivery. Expert Opin Drug Deliv 5: 385–401. 18426381 10.1517/17425247.5.4.385

[32] Jacobsen F, Hirsch T, Mittler D, Schulte M, Lehnhardt M, et al (2006) Polybrene improves transfection efficacy of recombinant replication-deficient adenovirus in cutaneous cells and burned skin. J Gene Med 8: 138–146. 16288494 10.1002/jgm.843

[33] Arcasoy SM, Latoche JD, Gondor M, Pitt BR, Pilewski JM (1997) Polycations increase the efficiency of adenovirus-mediated gene transfer to epithelial and endothelial cells in vitro. Gene Ther 4: 32–38. 9068793 10.1038/sj.gt.3300349 PMC7091802

[34] Schoehn G, El Bakkouri M, Fabry CM, Billet O, Estrozi LF, et al (2008) Three-dimensional structure of canine adenovirus serotype 2 capsid. J Virol 82: 3192–3203. 18216088 10.1128/JVI.02393-07 PMC2268473

[35] Doronin K, Toth K, Kuppuswamy M, Krajcsi P, Tollefson AE, et al (2003) Overexpression of the ADP (E3–11.6 K) protein increases cell lysis and spread of adenovirus. Virology 305: 378–387. 12573583 10.1006/viro.2002.1772

[36] Geisse S, Di Maiuta N, Ten Buren B, Henke M (2005) The secrets of transfection in serum-free suspension culture. In: Gòdia F, Fussenegger M, editors. Animal Cell Technology meets Genomics Springer Netherlands. 373–376.

[37] Alba R, Hearing P, Bosch A, Chillon M (2007) Differential amplification of adenovirus vectors by flanking the packaging signal with attB/attP-PhiC31 sequences: implications for helper-dependent adenovirus production. Virology 367: 51–58. 17560622 10.1016/j.virol.2007.05.014

[38] Clark PR, Stopeck AT, Brailey JL, Wang Q, McArthur J, et al (1999) Polycations and cationic lipids enhance adenovirus transduction and transgene expression in tumor cells. Cancer Gene Ther 6: 437–446. 10505854 10.1038/sj.cgt.7700074

[39] Chillon M, Alemany R (2011) Methods to construct recombinant adenovirus vectors. Methods Mol Biol 737: 117–138. 21590395 10.1007/978-1-61779-095-9_5

[40] Zabner J, Chillon M, Grunst T, Moninger TO, Davidson BL, et al (1999) A chimeric type 2 adenovirus vector with a type 17 fiber enhances gene transfer to human airway epithelia. J Virol 73: 8689–8695. 10482622 10.1128/jvi.73.10.8689-8695.1999 PMC112889

